# Prevention and Management of Heart Failure Associated with Type 2 Diabetics in Rural Australia

**DOI:** 10.3390/jcm15010304

**Published:** 2025-12-31

**Authors:** Allen G. Ross, Utpal K. Mondal, Shakeel Mahmood, Feleke H. Astawesegn, Anayochukwu E. Anyasodor, M. Mamun Huda, Subash Thapa, Setognal B. Aychiluhm, Santosh Giri, Md. Ferdous Rahman, Muhammad J. A. Shiddiky, Mohammad Ali Moni, Kedir Y. Ahmed

**Affiliations:** 1 College of Medicine, Ajman University, Ajman 346, United Arab Emirates; 2 Rural Health Research Institute, Charles Sturt University, Orange, NSW 2800, Australia; umondal@csu.edu.au (U.K.M.); shmahmood@csu.edu.au (S.M.); fastawesegn@csu.edu.au (F.H.A.); aanyasodor@csu.edu.au (A.E.A.); suthapa@csu.edu.au (S.T.); saychiluhm@csu.edu.au (S.B.A.); sgiri@csu.edu.au (S.G.); mdfrahman@csu.edu.au (M.F.R.); mshiddiky@csu.edu.au (M.J.A.S.); mmoni@csu.edu.au (M.A.M.); kahmed@csu.edu.au (K.Y.A.); 3 School of Rural Medicine, Charles Sturt University, Orange, NSW 2800, Australia; mhuda@csu.edu.au

**Keywords:** heart failure, type 2 diabetes, prevention, management, treatment, rural Australia

## Abstract

**Background:** Heart failure (HF) patients with a ‘reduced’ ejection fraction (HFrEF) have several proven treatment options, but for those with a ‘preserved’ ejection fraction (HFpEF) there are very few. However, recent trials such as the EMPEROR-Preserved and DELIVER have shown that sodium-glucose cotransporter 2 (SGLT2) inhibitors significantly reduce HF hospitalization in HFpEF patients, and these are now supported by both Australian and international guidelines. **Methods:** We undertook a narrative review using a structured multi-database search (MEDLINE, Embase, CINAHL, Scopus) and key Australian sources (AIHW, ABS, Department of Health and Aged Care) without geographic or publication-year restrictions. **Results:** In Australia there were approximately 179,000 hospitalizations in 2020–2021 due to HF equating to a rate of 697 per 100,000 population. The age-standardized hospitalization rate for HF in remote and very remote areas was 1.8 times higher than in major cities. Likewise, since 2000 the prevalence of diabetes has nearly tripled, from 460,000 to 1.3 million. In remote areas, there were 47,600 diabetes hospitalizations in 2021–2022, with residents being 2.5 times more likely to be hospitalized for diabetes compared to those in major cities. **Conclusions:** In rural Australia, reducing preventable hospitalizations and premature mortality from heart failure and type 2 diabetes requires a stronger rural generalist and general practitioner workforce, improved access to essential medicines and telehealth, and equity-focused evaluation.

## 1. Introduction

Heart failure (HF) is a major public health challenge worldwide and a leading cause of hospitalization and mortality, with a substantial burden among people with type 2 diabetes mellitus (T2DM) [1,2,3,4]. In Australia, the burden of HF continues to increase, driven by an aging population, rising obesity and increase in the prevalence of T2DM, which has tripled since 2000 [5,6,7]. Rural and remote populations experience disproportionately higher rates of HF-related hospitalizations and mortality, exacerbated by limited access to healthcare services and the complexities associated with managing comorbid conditions such as T2DM [6,8]. These differences are most evident among Aboriginal and Torres Strait Islander peoples, many of whom live in rural and remote regions [9]. Structural determinants, including the ongoing impacts of colonization, institutional racism, and unequal access to culturally safe, high-quality care, shape cardiovascular risk, timely diagnosis, uptake of evidence-based therapies, and outcomes for Aboriginal and Torres Strait Islander peoples [10]. In rural and remote settings, these determinants interact with geographic distance, workforce shortages, limited specialist and diagnostic capacity, travel and cost burdens, and variable digital connectivity, which also constrain access and continuity of care for non-Indigenous residents [9,10].

Therapeutic advances have improved outcomes for HF with reduced ejection fraction (HFrEF). However, effective treatment options for HF with preserved ejection fraction (HFpEF) remain limited [11,12,13]. Accordingly, the objective of this narrative review is to summarize the epidemiological burden and clinical impact of heart failure and type 2 diabetes in rural and remote Australia and to examine contemporary prevention and management strategies with proven cardiovascular and renal benefit. Moreover, we evaluated models of care relevant to rural and remote contexts, including culturally safe primary care and digitally enabled service delivery. The overarching aim is to identify practical implementation priorities and to inform policy for populations disproportionately affected by these conditions.

## 2. Methods

This narrative review was conducted through a comprehensive literature search of published studies and clinical guidelines related to the prevention and management of HF in individuals with T2DM, with a particular emphasis on rural and remote populations in Australia. Electronic databases, including MEDLINE (PubMed), Scopus, Embase were searched from database inception to December 2024. Google Scholar was consulted for citation tracking. Searches combined terms for heart failure (“heart failure”, “cardiac failure”, “cardiomyopathy”), diabetes (“type 2 diabetes”, “type 2 diabetes mellitus”, “T2DM”) and contextual factors (“rural”, “remote”, “Aboriginal and Torres Strait Islander”, “Indigenous”) using Boolean operators. No geographical restrictions and no lower limit on publication year were applied. In addition to peer-reviewed literature, publicly available documents (gray literature and policy) were sourced from key Australian organizations and repositories, including the Australian Institute of Health and Welfare (AIHW), the Australian Bureau of Statistics (ABS), the Department of Health and Aged Care, the National Indigenous Australians Agency/Closing the Gap, and the National Heart Foundation of Australia/Cardiac Society of Australia and New Zealand (NHF/CSANZ). Findings were synthesized narratively to identify evidence-based practices, highlight gaps in the literature, and derive implications for rural and remote health services. Current clinical guidance was reviewed from Diabetes Australia and the American Diabetes Association, alongside heart failure guidance from NHF/CSANZ and international societies, including the European Society of Cardiology (ESC) and the American Heart Association/American College of Cardiology/Heart Failure Society of America (AHA/ACC/HFSA), to ensure contemporary and relevant therapeutic practice is reflected.

## 3. Burden of Heart Failure in Rural Australia

**Figure 1:**
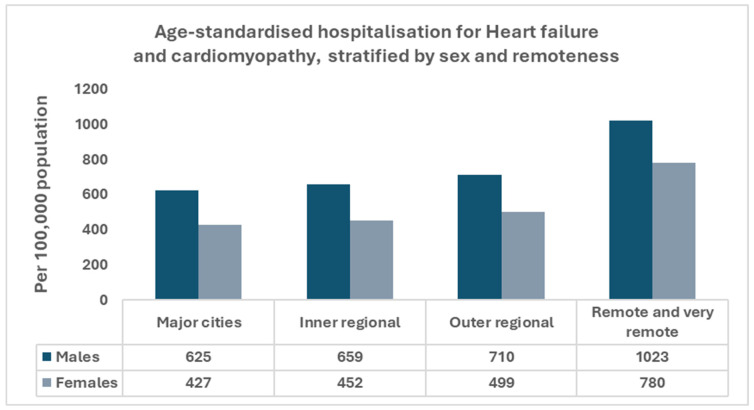
Heart failure and cardiomyopathy hospitalization rates by remoteness and sex, Australia 2020–2021. Age-standardized rates per 100,000 population are shown by remoteness category and sex, highlighting higher hospitalization rates in remote and very remote areas and consistently higher rates in males. Data source: Australian Institute of Health and Welfare [8,9].

In Australia, there were approximately 179,000 hospitalizations in 2020–2021 in which heart failure or cardiomyopathy was recorded as the principal and/or an additional diagnosis, equating to 697 per 100,000 population [8]. Hospitalization rates are higher in males than females after adjustment for age and increase steeply with advancing age, with the highest rates observed among people aged 85 years and over [8]. Australians living in rural and remote areas have a higher prevalence of cardiovascular risk factors, higher HF hospitalization rates, and higher mortality compared with people in metropolitan areas, with age-standardized heart failure hospitalization rates 1.8 times higher in remote and very remote areas than in major cities [9] (Figure 1). The burden is disproportionately higher among Aboriginal and Torres Strait Islander Peoples, particularly those residing in remote communities [14]. Consistent with these disparities, HF is classified as a chronic and potentially preventable hospitalization, underscoring the importance of timely primary care, optimization of chronic disease management, and coordinated follow-up, which are more difficult to maintain outside major cities [15,16]. Given the high potentially preventable admission load in rural and remote settings, prevention, optimization of guideline-based therapy, and continuity of care are priorities for reducing heart failure hospitalizations.

## 4. Burden of Type 2 Diabetes in Rural Australia

**Figure 2:**
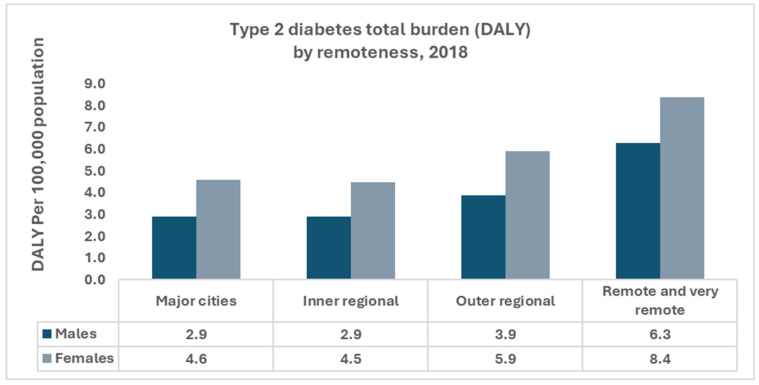
Type 2 diabetes burden in Australia, 2018. Disability-adjusted life years (DALYs) per 1000 population are shown by remoteness category and sex, demonstrating a higher burden in remote and very remote areas and among males. Data source: Australian Institute of Health and Welfare [17].

In 2021, over 1.3 million Australians (5.1%) were living with diabetes, and since 2000, the number of people with diabetes has nearly tripled, with approximately 60,000 new type 2 diabetes cases diagnosed annually [17]. In 2018, type 2 diabetes accounted for 124,000 years of healthy life lost (4.7 Disability-Adjusted Life Year [DALY] per 1000 population) and 2.2% of the total disease burden (Figure 2). In 2020–2021, diabetes responsible for $3.4 billion in health expenditure (2.3% of total), with type 2 diabetes comprising 68% of this amount [17]. Diabetes contributed to approximately 6000 deaths in 2022 and 21,900 deaths (11% of all deaths, when associated causes were included). In 2021–2022, there were 1.2 million hospitalizations (10% of all hospitalizations), in which diabetes was recorded as a principal and/or additional diagnosis [17]. Prevalence, hospitalization rates, mortality, and disease burden are higher among Aboriginal and Torres Strait Islander peoples, people living in lower socioeconomic areas, and residents of remote regions. Among First Nations people, type 2 diabetes accounted for 2.9% of the disease burden, 4.1 times higher than non-Indigenous Australians. In remote areas there were 47,600 diabetes-related hospitalizations in 2021–2022 and residents were 2.5 times more likely to be hospitalized for diabetes than those in major cities [9]. In view of the substantial and unevenly distributed burden of type 2 diabetes, particularly in rural and remote communities and among Aboriginal and Torres Strait Islander peoples, and its established contribution to HF incidence and hospitalizations, the next section outlines priorities for cardiometabolic risk reduction and early detection and considers integrated primary-care models appropriate for rural and remote practice.

## 5. Prevention of Heart Failure in Type II Diabetics

Heart failure (HF) patients with a ‘reduced’ ejection fraction (HFrEF) have a number of proven treatment options, but for those with a ‘preserved’ ejection fraction (HFpEF) there are very few [11,12,13]. It is, therefore, the responsibility of the primary-care physician to ensure that patients fully understand the necessity for an integrated, evidence-based approach (e.g., not just drugs) over the course of their lifetime [18,19,20]. A number of ‘SMART’ therapeutic goals have been put forward by the Australian and American Diabetic Associations based on recent clinical trials [11,12,13,18]. The evidence to date indicates that the dietary approaches to stop hypertension (DASH) or a Mediterranean diet that is low in carbohydrates, fat, salt (<6 g/d), and high in fiber is effective in lowering cholesterol, blood pressure, and maintaining blood glucose targets [21,22]. Patients are recommended to complete at least 150 min of moderate-intensity aerobic exercise (e.g., walk briskly) per week and, if possible, 2–3 sessions of resistance exercise (≥ 60 min/week) [18,23]. Younger diabetics could do a component of high-intensity interval training (HIIT) and in the elderly, yoga has shown benefits [24,25]. For patients who have never exercised or are extremely obese (BMI > 35 kg/m^2^) a graded program (e.g., starting with 10–15 min per day) should be commenced [18,23]. Even modest exercise has profound metabolic benefits [23]. For those overweight or obese, modest weight reduction (5–10%) has a significant impact on glucose control, cholesterol, renal function, and blood pressure [18,25]. However, most trials have demonstrated that after the initial weight loss, most weight is put back on within 1–2 years [26]. The body’s desire to return to its previous ‘physiological state’ is intense [26]. Alcohol consumption has been linked to both beneficial and adverse cardiovascular outcomes in observational research; however, these associations are subject to residual confounding and do not establish causality. Contemporary epidemiological evidence indicates that any cardioprotective effect of light-to-moderate alcohol intake is uncertain and outweighed by its adverse health consequences. Alcohol increases the risk of hypertension, atrial fibrillation, stroke, several cancers, and all-cause mortality, even at low levels of consumption [27,28,29]. Current public health guidance, including the Australian Guidelines to reduce health risks from drinking alcohol published by the National Health and Medical Research Council (NHMRC), emphasizes that no level of alcohol consumption is completely safe [29,30]. If alcohol is consumed, individuals should adhere to NHMRC low-risk recommendations, no more than ten standard drinks per week and no more than four on any one day [30]. Alcohol should not be recommended for the prevention of cardiovascular disease or type 2 diabetes. Smoking cessation is recommended for all patients with no level determined to be safe [31]. The blood pressure for diabetics has been set at <130/80 mm Hg, but for those >75 years of age the evidence suggests that a BP of <140/90 mm Hg may be appropriate [11,12,13,18]. The HbA1c target for diabetics is set at ≤7% but again for those over >75 years this may be relaxed to ≤8% given the increased rate of hypoglycemia and falls [11,12,13,18]. Cholesterol goals for diabetic patients are set at <4.0 mmol/L for total cholesterol; <2 mmol/L for LDL-C; ≥1 mmol/L for HDL-C; and Non HDL-C < 2.5 mmol/L [32]. Sleep apnea is strongly associated with HF, particularly among patients with reduced ejection fraction. Polysomnography is recommended for patients with symptoms such as snoring and obstructive sleep apnea [33]. As little as 4 h of continuous positive airway pressure (CPAP) per night has proven to be effective [33]. While CPAP therapy can improve sleep-related symptoms and quality of life in obstructive sleep apnea (OSA), large trials such as the SAVE study have not shown a reduction in cardiovascular outcomes [34]. Finally, it is also highly recommended that elderly patients receive annual influenza vaccination [35]. Prevention should be bidirectional: patients with heart failure require early metabolic risk management to avoid developing type 2 diabetes, and patients with diabetes need rigorous cardiovascular risk control to prevent heart failure [3,4].

Recognizing clinical or biochemical indicators of insulin resistance can help clinicians identify patients at higher cardiometabolic risk who are more likely to progress to type 2 diabetes or experience worsening heart failure [3,4]. Incorporating assessment of insulin resistance into routine heart failure care, particularly in primary care settings, may support earlier implementation of targeted lifestyle and pharmacological interventions that improve long term outcomes [18]. Ongoing monitoring of emotional well-being, medication adherence, weight, HbA1c, lipids, BP, and renal function are required to prevent HF [20,36].

## 6. Pharmacological Management of Heart Failure in Type 2 Diabetics

Pharmacological therapies for heart failure are broadly categorized into two groups: (1) disease-modifying treatments that improve prognosis by reducing mortality and hospitalizations, and (2) symptomatic therapies that alleviate congestion without altering long-term outcomes. In HFrEF, the core ‘pillars of therapy’ as endorsed in Australian national guidelines include renin–angiotensin system inhibitors (ACE inhibitors or ARNI), beta-blockers, mineralocorticoid receptor antagonists, and sodium-glucose cotransporter 2 (SGLT2) inhibitors, all of which are recommended for all eligible patients [37,38]. In contrast, loop diuretics such as furosemide are used to manage volume overload and symptoms but have no prognostic benefit [38]. For HFpEF, SGLT2 inhibitors have recently emerged as the first pharmacologic option to reduce heart failure events, while diuretics remain important for symptom control [37,39]. Experimental agents discussed later in this manuscript, such as vericiguat and omecamtiv mecarbil, are not yet included in national treatment recommendations and should be considered investigational.

### 6.1. HFrEF Patients

As mentioned, HF patients with HFrEF have a number of proven treatment options [12]. If a HFrEF patient has a problem with congestion, *Loop Diurectics* (e.g., furosemide, bumetanide, and torsemide) are preferred [12]. The bioavailability of furosemide is reduced compared to bumetanide and torsemide thus they are preferred for right-sided HF [12]. Metolazone, a *thiazide-like diuretic*, is used in combination with furosemide in patients who become resistant to loop diuretics [12]. Angiotension converting enzyme inhibitors (*ACEi*) (e.g., ramipril, captopril) or Angiotension receptor blockers (*ARB*) (e.g., valsartan, candesartan) are recommended for HFrEF patients with cardiovascular disease and mild (41–49%) LVEF reduction in order to decrease hospitalization and mortality [40,41]. Both the AIRE and CHARM trial follow-ups support their clinical application in patients with HFrEF [40,41]. Sacubitril/valsartan (angiotensin receptor–neprilysin inhibitor [ARNI]) is an established, guideline-recommended therapy for HFrEF [42]. It is a first-in-class angiotensin receptor neprilysin inhibitor (*ARNI*), combining inhibition of RAAS and potentiation of the counter-regulatory natriuretic peptide system [42]. The PARADIGM-HF trial revealed that LCZ696 was superior to enalapril in reducing the risks of death and hospitalization for HF [42]. In symptomatic HFrEF (LVEF ≤ 40%) who tolerate renin–angiotensin system blockade, ARNI is preferred over ACEi/ARB to reduce cardiovascular death and HF hospitalization; when switching from an ACE inhibitor, a 36 h washout is required [42]. *Hydralazine/isosorbide dinitrate* may be used in HFrEF patients that are not able to tolerate a ACEi or ARB [43,44]. It has also shown to be of benefit in African patients with NYHA class III or IV symptoms [43,44]. Mineralocorticoid receptor antagonist (*MRAs*) (e.g., spironolactone, eplerenone), and *4 beta-blockers* (e.g., carvedilol, bisoprolol, metoprolol succinate, nebivolol) are recommended for HFrEF patients with moderate to severe (≤40%) LVEF reduction to decrease hospitalization and mortality [43,44]. A substantial body of evidence has shown that *Metformin*, ‘the old kid on the block’, is at least as safe as other glucose-lowering agents in patients with a reduced LVEF or concomitant chronic kidney disease [45,46]. Thus, it continues to be recommended as the first-line glycemic agent globally.

Sodium–glucose co-transporter-2 inhibitors (*SGLT2*) (e.g., empagliflozin, dapagliflozin), are another new ‘kid on the block’ that have generated global interest [47,48,49,50,51,52,53,54]. As a result, they are recommended as a second-line treatment for glycemic control after metformin in people with HF [47,48,49,50,51,52,53,54]. The EMPA-REG OUTCOME trial, DAPA-HF trial, CVD-REAL trial, EMPEROR-Reduced and the VERTIS CV trial, have all shown that SGLT2 lowers the risk of HF, renal impairment, and overall mortality [47,48,49,50,51,52,53,54]. There may be a role for *ARNI/SGLT2 inhibitor therapy* in high-risk HF patients [55]. The DAPA-HF trial found that dapagliflozin was efficacious and safe in patients who were and who were not taking LCZ696 which suggests that the use of both agents together could further lower morbidity and mortality in patients with HFrEF [55]. SGLT2 inhibitors exert important metabolic effects, including reductions in insulin resistance, improved energy utilization, and lower glucose related stress. These metabolic changes are increasingly recognized as contributing to the reductions in heart failure events observed in major trials, including among people without diabetes [3,4,52]. Glucagon-like peptide-1 receptor agonists (*GLP-1RA*) (e.g., semaglutide, liraglutide) may also be of some benefit for patients with HF but their predominate role is in lowering the risk of stroke and peripheral artery disease and only modestly improving LVEF in HF patients [55,56,57,58,59,60,61]. *Ivabradine* is reserved for patients with a LVEF ≤ 35 and sinus rate of 70 beats per minute when both an ACEi and beta blocker were ineffective [62]. The SHIFT trial supports the importance of heart-rate reduction with ivabradine [62]. *Digoxin* may be considered for patients with HFrEF in sinus rhythm who remain symptomatic (NYHA III–IV) despite maximally tolerated guideline-directed medical therapy (ACEi/ARB or ARNI, a β-blocker, and an MRA), primarily to reduce recurrent heart-failure hospitalization and improve symptoms; it does not confer a survival benefit [43,44].

In rural and remote Australia, the selection and effectiveness of pharmacological therapies for patients with type 2 diabetes and heart failure are shaped by healthcare access, comorbidity burden, kidney function, capacity for monitoring, and medication adherence. SGLT2i, particularly empagliflozin and dapagliflozin, are suited to these contexts because they confer cardio-renal benefit, are orally administered, and have a low risk of hypoglycemia; for patients facing food insecurity or limited self-monitoring, they are often safer than sulfonylureas [63,64]. These attributes are particularly relevant where chronic kidney disease is common and specialist services are scarce [63]. Conversely, thiazolidinediones (e.g., rosiglitazone) are generally avoided because of their association with fluid retention and worsening heart failure, especially where monitoring of volume status and renal function is limited [65]. For Aboriginal and Torres Strait Islander peoples living with diabetes, who frequently experience higher rates of obesity, insulin resistance, and cardiovascular risks such as GLP-1 receptor agonists (e.g., semaglutide) may offer additional benefit through weight reduction and cardiovascular risk mitigation; however, uptake can be constrained by cost, cold-chain requirements, and injectable administration in remote communities [66]. Therapeutic choices should therefore be tailored to local health-system capacity and patient circumstances, using shared decision-making and continuity of care within primary-care led models.

### 6.2. HFpEF Patients

Only two HFrEF drugs (e.g., MRAs, ARNI) have shown to be of some benefit in HFpEF patients [43,44]. But recent evidence from large-scale trials has confirmed that SGLT2 inhibitors significantly reduce the risk of heart failure hospitalization in patients with HFpEF. The EMPEROR-Preserved and DELIVER trials demonstrated that empagliflozin and dapagliflozin, respectively, are effective in improving clinical outcomes in this group, regardless of diabetes status [39,67]. These findings are reflected in updated Australian guidelines, including the 2022 consensus statement by Sindone et al., which recommends early initiation of both ARNI and SGLT2 inhibitors in eligible patients. SGLT2 inhibitors have also been added to the PBS with a specific indication for HFpEF [37]. There is some evidence that *Omega-3 PUFA supplementation* is reasonable to use as adjunctive therapy in patients with NYHA class II–IV symptoms and HFrEF or HFpEF to reduce mortality and hospitalization [43,44]. Emerging data also suggest a potential role for GLP-1 receptor agonists in HFpEF, particularly among patients with obesity. The STEP-HFpEF trial showed that semaglutide improved functional capacity and quality of life in obese HFpEF patients, although further research is needed before guideline recommendations can be made [68].

Some new ‘infants on the block’ for HF include *Vericiguat*, a novel oral soluble guanylate cyclase simulator, that showed improvement in quality of life in patients with HFpEF in the SOCRATES-PRESERVED trial [69,70]. *Amipretide*, a novel mitochondrial modulating agent, improves myocardial energetics [71]. However, the PROGRESS-HF Phase 2 trial showed that elamipretide was well tolerated but did not improve LVESV at 4 weeks in patients with stable HFrEF compared with placebo [71]. Further phase 3 trials are needed. The effects of the cardiac myosin activator, *Omecamtiv-mecarbil*, has been recently examined in the rat model to determine its effects on severe chronic aortic regurgitation [72]. The drug exerted favorable hemodynamic effects in rats [72]. The next step will be to assess the drug in the primate model. Finally, the following drugs should be avoided in HF patients: NSAIDS, thiazolidinediones, diltiazem, verapamil, and moxonidine [65,73]. Nondihydropyridine calcium channel blockers have negative inotropic effects that may be harmful in asymptomatic HF patients with low LVEF [43,44]. α-Adrenergic blockers such as doxazosin should also be avoided [43,44].

## 7. Advancing Heart Failure Care in Rural Australia

Despite significant advancements in the prevention and treatment of cardiovascular diseases (CVD), the burden of heart failure is anticipated to rise in Australia due to an aging population with increasing rates of obesity [74,75,76]. It is, therefore, crucial to implement a holistic approach to improve health outcomes and reduce the healthcare burden of heart failure among rural and remote Australians, who face an elevated risk compared to urban populations [38,74,77]. This approach should prioritize early diagnosis, enhanced screening programs, improved self-management, and community-based management through primary-care to significantly enhance health outcomes and minimize disease severity [77,78,79]. General practitioners (GPs) are central to the prevention, diagnosis, and ongoing management of heart failure across metropolitan, regional, and remote Australia. As the first point of contact and principal providers of ongoing care, they diagnose HF, assess each patient’s risk of complications such as hospitalization or death, start and adjust guideline-recommended medicines to effective (or maximally tolerated) doses, and coordinate multidisciplinary management across cardiac, renal, and diabetes care. When advanced intervention is required, they ensure timely referral and continuity between primary and specialist services [38,80,81,82,83]. In regional and remote areas, rural generalists frequently lead hospital and emergency services and manage GP-run facilities, maintaining continuity across inpatient and community settings. Where available, they partner with cardiology through shared-care and telehealth to sustain access to contemporary therapies. These roles are recognized in national guidance and implementation reports that emphasize GP-centered models of care and support for optimization of life-prolonging therapies in primary care [38,80,81,82,83]. Within this framework, self-care and risk-factor modification remain fundamental to preventing incident of HF and improving outcomes. Priorities include heart-healthy dietary patterns, regular physical activity, smoking cessation, and weight management; optimization of glycemic control, blood pressure, and lipids; and the judicious use of evidence-based pharmacotherapies with cardiovascular benefit [18,21,22,23,24,25,77].

To improve the effectiveness of conventional HF strategies among individuals with type 2 diabetes (T2DM), particularly in rural and remote Australia, targeted modifications are needed. First, systematic screening for subclinical cardiac dysfunction in T2DM patients using natriuretic peptides or echocardiography should be integrated into routine primary care to enable early intervention before overt HF develops [3]. Second, multidisciplinary care models involving general practitioners, diabetes educators, and nurse practitioners can enhance treatment coordination across glycemic, cardiac, and renal domains [80]. Third, pharmacologic regimens should align evidence-based HF therapies (e.g., ACE inhibitors, beta-blockers) with glucose-lowering agents that confer cardiovascular benefits, such as SGLT2 inhibitors or GLP-1 receptor agonists, while considering renal safety and comorbidities [3,84]. Finally, tailoring lifestyle interventions and self-management education to the cultural and logistical realities of rural communities especially Aboriginal and Torres Strait Islander populations can improve engagement and adherence [80].

Improving diagnosis and management for patients with both T2DM and HF in rural and remote areas requires a structured and context-specific approach. An optimal model would begin with structured screening in primary care using validated tools to assess HF symptoms (e.g., dyspnea, fatigue, edema) alongside metabolic parameters such as HbA1c and eGFR. Where feasible, point-of-care testing and portable echocardiography may be incorporated. Clinical data can then be securely transmitted via telehealth platforms to multidisciplinary teams in regional or metropolitan centers for remote evaluation and collaborative care planning. The use of wearable devices for continuous glucose and heart rhythm monitoring can enhance early detection of deterioration. This approach supports timely intervention and improves continuity of care, especially in geographically isolated populations. Importantly, it aligns with national digital health frameworks and emerging models shown to be feasible in Indigenous and non-Indigenous rural communities [85,86].

Despite these interventions, managing heart failure in Australia remains challenging as many patients with cardiovascular complications related to T2D are diagnosed at an advanced stage, often due to limited access to healthcare services and a lack of awareness of the early signs [77,79,80,82]. To address this, robust screening programs should be established in rural and remote communities [38,82]. Primary healthcare providers should receive training to detect cardiovascular complications and undiagnosed T2D at earlier stages [77,79,80,82]. Effectively reaching rural populations can be achieved through community health clinics, GP clinics, and partnerships with local non-governmental organization (NGOs). Collaborative efforts involving primary healthcare providers, diabetes specialists, and cardiac care teams ensure coordinated treatment plans across different levels of care [38,77,79,80,81,82,83]. Establishing cardiac centers and diabetes clinics in larger rural regional towns is of utmost importance, as these facilities can serve as educational hubs, monitoring centers, and specialized care providers. Expanding the utilization of digital health and telemedicine services can extend access to advanced care, facilitating disease monitoring and remote consultations, thereby reducing cardiovascular-related hospitalizations and mortality risks among heart failure patients [82,87]. Empowering patients and their families to manage cardiovascular disease in their local communities is a critical strategy, with primary healthcare workers playing a pivotal role through home visits, care coordination, and treatment adherence support, especially for elderly patients and those with impaired mobility [38,77,82]. Additionally, advocating for policy changes addressing healthcare disparities in rural and remote Australia and increasing funding for the recruitment of diabetes specialists and cardiologists to larger regional centers will be paramount to ensuring comprehensive continuum of care.

## 8. Conclusions

The growing burden of HF and T2D in rural and remote Australia requires targeted, context specific action. Integrated models led by primary care that include early detection, culturally safe self-management support, and pharmacotherapy recommended by clinical guidelines are central to reducing avoidable admissions and premature mortality. For HFrEF, routine use and systematic titration of the four therapeutic classes (angiotensin-converting enzyme inhibitor or angiotensin receptor–neprilysin inhibitor, beta-blocker, mineralocorticoid receptor antagonist, and sodium–glucose cotransporter-2 inhibitor) should be standard where indicated. For HFpEF, sodium–glucose cotransporter 2 inhibitors should be incorporated to reduce heart failure events in eligible patients. GPs and rural generalists coordinate care across cardiac, renal, and metabolic services; telehealth and shared care extend specialist input while maintaining continuity within local services. Policy priorities include resourcing culturally safe services, particularly for Aboriginal and Torres Strait Islander communities, strengthening the rural generalist workforce, developing regional hubs with outreach diagnostics, improving the availability and affordability of essential medicines, and investing in digital connectivity. Implementation should include explicit equity objectives and prospective evaluation to demonstrate improvements in access, quality of care, and patient outcomes.

## Data Availability

The original contributions presented in the study are included in the article, further inquiries can be directed to the corresponding author.
